# Ambipolar ferromagnetism by electrostatic doping of a manganite

**DOI:** 10.1038/s41467-018-04233-5

**Published:** 2018-05-15

**Authors:** L. M. Zheng, X. Renshaw Wang, W. M. Lü, C. J. Li, T. R. Paudel, Z. Q. Liu, Z. Huang, S. W. Zeng, Kun Han, Z. H. Chen, X. P. Qiu, M. S. Li, Shize Yang, B. Yang, Matthew F. Chisholm, L. W. Martin, S. J. Pennycook, E. Y. Tsymbal, J. M. D. Coey, W. W. Cao

**Affiliations:** 1 Condensed Matter Science and Technology Institute, School of Science, Harbin Institute of Technology, Harbin, 150081 China; 2 School of Physical and Mathematical Sciences & School of Electrical and Electronic Engineering, Nanyang Technological University, Singapore, 639798 Singapore; 3 Department of Materials Science and Engineering, National University of Singapore, Singapore, 117575 Singapore; 4 Department of Physics and Astronomy & Nebraska Center for Materials and Nanoscience, University of Nebraska, Lincoln, Nebraska 68588 USA; 5 School of Materials Science and Engineering, Beihang University, Beijing, 100191 China; 6 NUSNNI-NanoCore, National University of Singapore, Singapore, 117411 Singapore; 7 Department of Materials Science and Engineering, University of California, Berkeley, Berkeley, CA 94720 USA; 8 Materials Sciences Division, Lawrence Berkeley National Laboratory, Berkeley, CA 94720 USA; 9 School of Materials Science and Engineering, Harbin Institute of Technology, Shenzhen, Guangzhou 518055 China; 10 Shanghai Key Laboratory of Special Artificial Microstructure Materials and Technology & Pohl Institute of Solid State Physics & School of Physics Science and Engineering, Tongji University, Shanghai, 200092 China; 11 Materials Science and Technology Division, Oak Ridge National Laboratory, Oak Ridge, TN 37831 USA; 12 School of Physics, Trinity College, Dublin, 2 Ireland; 13 Faculty of Materials Science and Engineering, Beihang University, Beijing, 100191 China; 14 Department of Mathematics and Materials Research Institute, The Pennsylvania State University, University Park, PA 16802 USA

## Abstract

Complex-oxide materials exhibit physical properties that involve the interplay of charge and spin degrees of freedom. However, an ambipolar oxide that is able to exhibit both electron-doped and hole-doped ferromagnetism in the same material has proved elusive. Here we report ambipolar ferromagnetism in LaMnO_3_, with electron–hole asymmetry of the ferromagnetic order. Starting from an undoped atomically thin LaMnO_3_ film, we electrostatically dope the material with electrons or holes according to the polarity of a voltage applied across an ionic liquid gate. Magnetotransport characterization reveals that an increase of either electron-doping or hole-doping induced ferromagnetic order in this antiferromagnetic compound, and leads to an insulator-to-metal transition with colossal magnetoresistance showing electron–hole asymmetry. These findings are supported by density functional theory calculations, showing that strengthening of the inter-plane ferromagnetic exchange interaction is the origin of the ambipolar ferromagnetism. The result raises the prospect of exploiting ambipolar magnetic functionality in strongly correlated electron systems.

## Abstract (web summary)

An ambipolar ferromagnet with both electron- and hole-doped ferromagnetism in a single material would facilitate understanding of ferromagnetic semiconductors for spintronic applications. Here the authors demonstrate ambipolar ferromagnetism in LaMnO_3_, using ionic liquid gating enabled electrostatic doping to produce electron–hole asymmetry.

## Introduction

Manipulating carriers of either sign and flipping the orientation of ferromagnetically coupled spins are operations that are basic to current information technology. An ambipolar ferromagnet that would allow the ordered spins to coexist with either electrons or holes in a single material, without modifying its crystal structure or chemical composition, is an appealing prospect. It could lead to advances in fundamental understanding of magnetic semiconductors and form a building block for new electric-field controlled spintronics based on the ability to create, control and detect the spin-polarized holes or electrons. Operating locally and reversibly, the electric-field offers notable advantages over magnetic field or hard chemical doping in the fabrication and operation of spintronic devices. Recently, there has been a lot of interest in ambipolar functionality, such as ambipolar conductivity in graphene^1^, black phosphorus^2^, and organic materials^3^. However, ferromagnetic semiconductors have so far been based on either electron-doped or hole-doping, but never both in the one material, precluding possible ambipolar spintronic devices, such as a dual-channel field-effect spin-filter or a field-effect spin valve.

Researchers are exploring various ways to create amipolar functionality in materials. Electrostatic doping^4,5^ of the mixed-valence manganites^6^ using an ionic liquid gate is a promising approach to try to realize ambipolar ferromagnetism, in light of the manganites’ rich magnetic phase diagrams and the possibility of generating high carrier densities of up to ~10^15^ cm^−2^, using an ionic liquid as gate dielectric. Ionic liquids have been used to achieve various other effects, including ambipolar doping layered materials^2^, superconductivity^4,7–14^, charge density waves^15^, metal–insulator transitions,^16,17^ and large resistive responses in mixed-valence manganites^18–21^. However, ambipolar ferromagnetism has proved difficult to achieve, owing to the challenge of varying the carrier density over a wide range while controlling the carrier–spin interactions. Nonetheless, from a material’s point of view, as an end member of several mixed-valence manganite solid solutions, LaMnO_3_ (LMO) is a good candidate for the realization of ambipolar ferromagnetism. The manganese is trivalent (Mn^3+^; 3*d*^4^) in bulk LMO, which exhibits an A-type antiferromagnetic ground state, but offers possibilities for hosting ferromagnetism with both electron (Mn^2+^; 3*d*^5^) and hole (Mn^4+^; 3*d*^3^) doping^22,23^.

Here we have managed to achieve ambipolar ferromagnetism by electrostatically gating atomically thin LMO films using an ionic liquid as a gate dielectric.

## Results

### Basic characterization

**Fig. 1:**
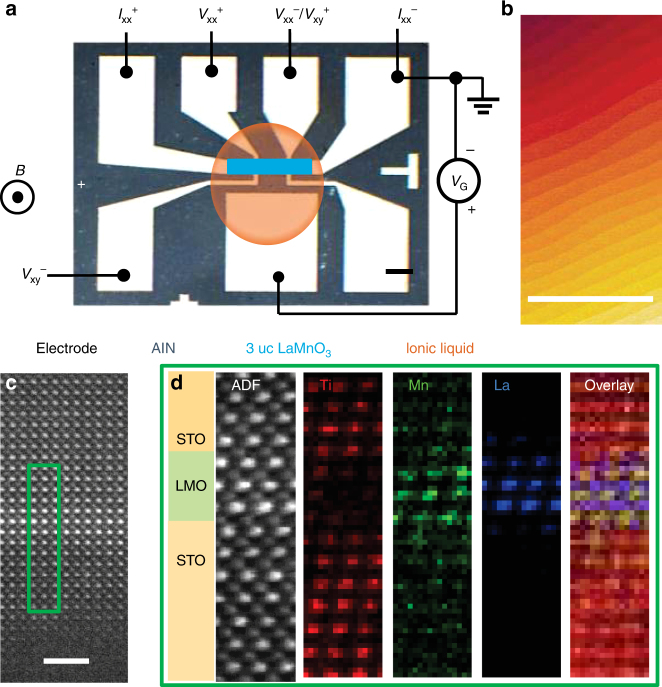
Schematic and structural characterization of the electric double-layer transistor. **a** Schematic of the device, fabricated by ultraviolet lithography. The exposed area of LaMnO_3_ is 300 µm in length and 50 µm in width. The scale bar is 100 µm. **b** Atomically flat surface of 3 unit cell (uc) LaMnO_3_ film grown on a SrTiO_3_ (001) substrate characterized by atomic force microscopy. The scale bar is 1 µm. **c** High-angle annular dark field (HAADF) image of the SrTiO_3_-capped sample. The scale bar is 2 nm. **d** EELS of the SrTiO_3_-capped sample. The corresponding EELS region is indicated in the green box in the HAADF image (**c**)

**Fig. 2:**
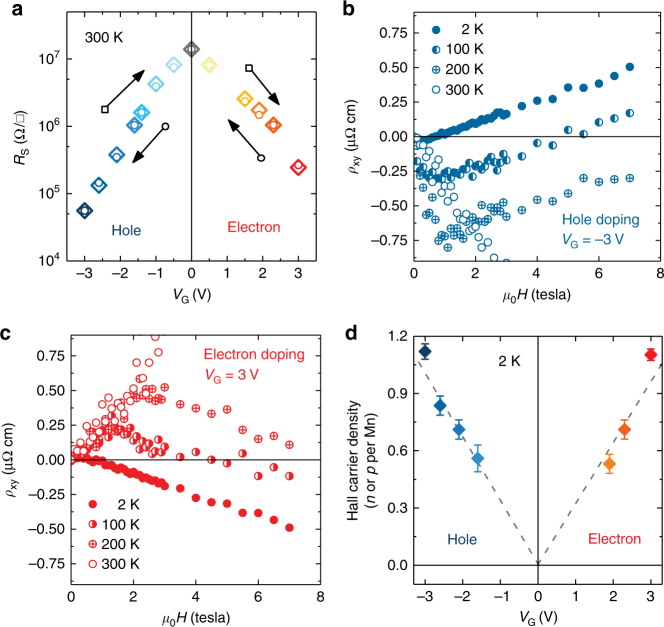
Transport properties of 3 uc LaMnO_3_ film at various gate voltages (*V*_G_) and temperatures. **a** The sheet resistance as a function of *V*_G_ at 300 K. No loop is seen, indicating that effects of oxygen migration are negligible. **b**, **c** Hall resistivity (*ρ*_*xy*_) of LaMnO_3_ under of *V*_G_ = −3 V (**b**) and 3 V (**c**) at different temperatures. temperatures. Due to the presence of high noise levels in *ρ*_*xy*_ (300 K) for magnetic field above 3 Tesla, the high-field data of *ρ*_*xy*_ at 300 K are omitted. **d** Hall carrier density expressed in electron or hole content per Mn site based on the Hall results at 2 K and various *V*_G_. The error bars at these *V*_G_ were calculated based on the fluctuation of *ρ*_*xy*_ within the ordinary Hall effect (OHE) region at 2 K

Figure 1a shows the electric double-layer transistor (EDLT) with a planar device configuration fabricated on a three unit cell (uc) thick LMO film^24^ grown on SrTiO_3_ (001) (STO) with an ionic liquid as the electrolyte (Methods section). Figure 1b illustrates the atomically flat surface of LMO after the gating measurements, revealed by an atomic force microscope. The high quality surface after gating indicates that etching of the material due to ionic liquid gating is negligible. In addition, we performed high-resolution cross-section scanning transmission electron microscopy (STEM) and electron energy loss spectroscopy (EELS) on a 3 uc LMO sample with an overlayer of STO. The STO overlayer was added in order to avoid damage during the STEM sample preparation to correctly characterize the interface between LMO and STO (see Supplementary Note 7). Figure 1c shows a high-angle annular dark field (HAADF) image of the sample along the [010] zone axis. Figure 1d shows the EELS results of the region of green square in Fig. 1c, demonstrating that the extent of intermixing at the LMO-STO interface is limited to 1 uc. The ultrathin layer thickness is a prerequisite for our study, because the required modulation of the carrier density is very large. In thick films, the induced carriers will be spread out, resulting in little modulation of the density. Furthermore, the surface and interface states in ultrathin films are more sensitive to the external modulations, such as electrostatic doping^25,26^. Carrier doping was realized by applying a gate voltage (*V*_G_) to the double-layer capacitor formed by the ionic liquid. The sheet resistance (*R*_S_) of the LMO film at 300 K decreases linearly on both sides of zero-voltage, as shown in Fig. 2a. This gating response is typical of an ambipolar material. It is important to note that no hysteresis is observed while sweeping *V*_G_, demonstrating that diffusion of oxygen vacancies is negligible during the gating experiments.

### Electrostatic gating

In order to further investigate the carrier behavior and the intrinsic magnetic properties of the material, the Hall resistivity (*ρ*_*xy*_) of the 3 uc LMO film was measured at different temperatures for *V*_G_ = ± 3 V. The Hall effect in a ferromagnet is a combination of the ordinary Hall effect (OHE) and the anomalous Hall effect (AHE)^27,28^. In ferromagnetic mixed-valence manganites, the OHE and AHE contributions to *ρ*_*xy*_ have opposite trends, resulting in an anomalous *ρ*_*xy*_ behavior different from that in ordinary ferromagnetic materials. The AHE starts to dominate above ~100 K, increases sharply around the Curie temperature (*T*_C_), peaking at a temperature roughly 30 K above *T*_C_, and decreases slowly at higher temperatures^29^. Figure 2b, c shows *ρ*_*xy*_ of LMO at various temperatures in the electron-doped and hole-doped regimes, exhibiting an AHE which is in good agreement with studies on La_1−*x*_A_*x*_MnO_3_ (A = Ba, Ca, Pb, Ce, and Sr) single crystals^23,30,31^. Typically, the AHE in hole-doped manganites, such as La_1−*x*_Sr_*x*_MnO_3_ and La_1−*x*_Ca_*x*_MnO_3_, is prominent^31^ at temperatures close to the Curie temperature, *T*_C_, namely in the higher temperature region of the metallic phase, where d*ρ*_*xx*_/d*T* is positive and the lower temperature region of the insulating phase, where d*ρ*_*xx*_/d*T* is negative. When the temperature is well below or above the *T*_C_, the Hall resistance becomes linear in magnetic field. These generic features are consistent with our experimental observation. The maximum magnitude of the AHE for La_1−*x*_Sr_*x*_MnO_3_ and La_1−*x*_Ca_*x*_MnO_3_ are ~0.65 and 1.3 µΩ cm, which is also consistent with the magnitude of the AHE in our study. Since the AHE is due to broken time-reversal symmetry, typically as a result of spin–orbit coupling in a ferromagnetic phase^28,32,33^, the data demonstrate the ferromagnetic nature of the films under both positive and negative gating.

The carrier densities of the electrons and holes under different gate voltages were calculated from the Hall measurement at 2 K, and are shown in Fig. 2d with *n*(*p*) representing the electron(hole) content per Mn. At 2 K, *ρ*_*xy*_ exhibits a linear variation with magnetic field (*μ*_0_*H*), and opposite signs of the slope are observed according to the sign of *V*_G_, revealing electron and hole characteristics of the LMO under positive and negative gate voltages, respectively. However, the values of carrier density deduced at 2 K are generally much larger than the nominal doping concentration^23,28,30,31^, although the sign of the slope of *ρ*_*xy*_ at low temperature is correct in our study, where the Hall carrier density of doped LMO achieved at ±3 V corresponds to roughly one electron or hole per Mn site. The abnormally large Hall carrier density of manganites is well documented in the existing literature^23,28,30,31^, but its mechanism remains unexplained. For instance, nominal chemically doped La_1−*x*_*A*_*x*_MnO_3_ thin films with ~0.1e per Mn always exhibit roughly one, or even more, electrons or holes per Mn site in Hall measurements at ~ 2 K. Nevertheless, a linear modulation of carrier density per Mn is confirmed. As shown in Fig. 2d, the Hall carrier density, which was estimated based on the normal Hall resistance regime of *ρ*_*xy*_, shows that the gating effect on carriers is roughly symmetric on both sides. Although the doping effect is often found to be asymmetric due to the difference in the size of the positive and negative ions in the liquid, the capacitance for electron-doped and hole-doping can also be symmetric in some cases^34,35^.

### Ambipolar ferromagnetism

**Fig. 3:**
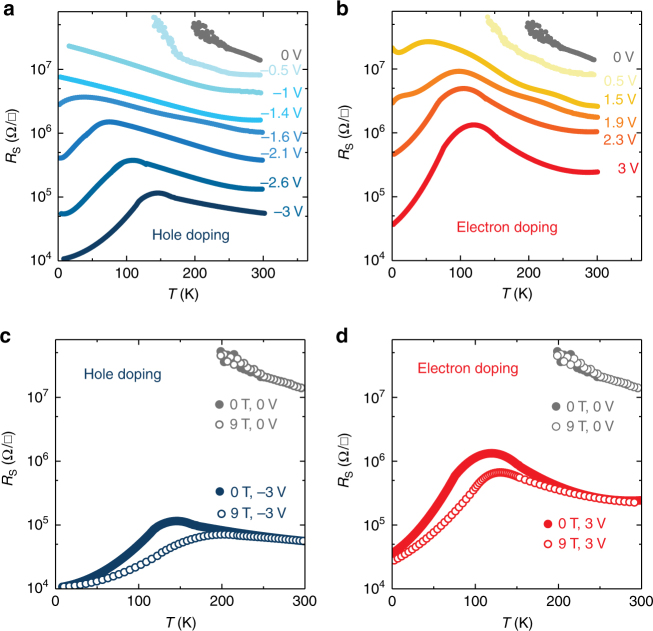
Metal–insulator transition (MIT) in ionic liquid-gated 3 uc LaMnO_3_. **a**, **b** The sheet resistance as a function of gate voltages. Both negative (**a**) and positive (**b**) voltage regimes show a MIT, accompanying the reduction of sheet resistance. **c**, **d** Temperature dependence of magnetoresistance in the hole-doped and electron-doped 3 uc LaMnO_3_ film

The realization of ambipolar ferromagnetism is further confirmed by the variation of studies of sheet resistance *R*_S_ as a function of temperature for various gate voltages. Figure 3a, b show the *R*_S_ vs. temperature curves of the 3 uc LMO under positive or negative *V*_G_. By increasing the negative *V*_G_ (hole accumulation, Fig. 3a), the *R*_S_ vs temperature curves exhibit a metal–insulator transition (MIT) with a resistivity peak at a temperature *T*_P_, accompanying the decrease of resistivity. The same trend is observed when *V*_G_ is positive (electron accumulation, Fig. 3b). Below *T*_P_, electron-doped and hole-doped LMO both exhibit metallic behavior. The sheet resistance of our samples are quantitatively consistent with the resistance quantum (*h*/*e*^2^ = 25.8 kΩ), and the well-documented MIT in manganite. In the temperature regime above the MIT, the system is non-magnetic and enters a semiconducting state. This is consistent with all understood cases with a resistance greater than *h*/*e*^2^. In the temperature regime near and below the MIT, the resistivity of our sample is quantitatively consistent with the well-documented resistivity of manganite materials. For example, the resistivity of our samples at the peak temperature is ~10^−1^ to 10^−2^ Ωcm, showing the same value as the reported resistivity at the peak temperature *T*_P_^22,36^. Furthermore, *T*_P_ in both electron-doped and hole-doped regimes increases upon applying a 9 Tesla magnetic field (Fig. 3c, d), and colossal magnetoresistance (CMR) is observed. The field-induced increase of *T*_P_ resembles that of all other chemically doped ferromagnetic manganites, proving that the ferromagnetic metallic state induced by both positive and negative gating lies within LMO films, rather than at the surface of the STO substrate^37^.

### Electron–hole asymmetry

**Fig. 4:**
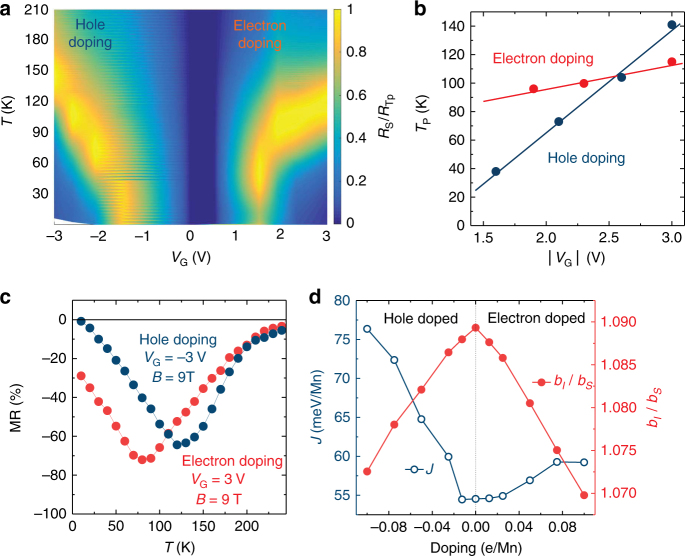
Electron–hole asymmetry in electrostatically gated LaMnO_3_. **a** Contour plot of sheet resistance (*R*_S_) as a function of gate voltage (*V*_G_) and temperature. **b** Evolution of the resistivity peak (*T*_P_) of *R*_S_ in the electron-doped and hole-doped regimes. **c** Asymmetric peak magnitude and temperature of the magnetoresistance (MR), defined as (*R*(*H*)−*R*(0))/*R*(0)) (%), for an electron-doped or hole-doped LaMnO_3_ film. **d** First-principles calculations on the magnetism of LaMnO_3_ under bipolar gating. The exchange coupling parameter and *J* (open blue dots) and the distortion parameter *b*_*l*_/*b*_*s*_ (filled red dots) are shown as a function of doping

Strikingly, the doping dependence of *T*_P_ for electron doping is less sensitive than that for hole doping. Figure 4a presents a contour plot of normalized sheet resistance (*R*_S_(*T*)/*R*_*T*p_) vs temperature and *V*_G_. The contour plot shows that *T*_P_ increases asymmetrically for positive and negative gating. Figure 4b summarizes the data on *T*_P_ as a function of *V*_G_, demonstrating the electron–hole asymmetry of the CMR, and of the ferromagnetic ordering temperature. The electron–hole asymmetry is further confirmed by the asymmetric magnitude and characteristic temperature of the temperature-dependent magnetoresistance (MR), defined as (*R*(*H*)−*R*(0))/*R*(0) (%), for electron-doped and hole-doped LMO film (Fig. 4c).

### Density functional theory calculations

First-principles density function theory (DFT) calculations were employed to understand the origin of the asymmetric ambipolar ferromagnetism (see Supplementary Note 6 for details). The magnetism in LMO can be described by an isotropic exchange Hamiltonian, $H = - \mathop {\sum }\limits_{ < ij > } J_{ij}S_i.S_j$, where *J*_*ij*_ is the exchange interaction between neighboring Mn ions with localized spin moments *S*_*i*_ and *S*_*j*_. The intra-plane and inter-plane exchange interactions have different values. The interplay of intra-plane *J*_*ab*_ and inter-plane *J*_*c*_ determines overall magnetic ordering (Supplementary Note 6). In fully relaxed bulk LMO, the intra-plane exchange *J*_*ab*_ is ferromagnetic, whereas the inter-plane exchange *J*_*c*_ is antiferromagnetic, which leads to the A-type antiferromagnetic ordering^38^. In the biaxially strained epitaxial films, we find that both exchange interactions are ferromagnetic, which results in overall ferromagnetic ordering. In the mean-field approximation, the strength of ferromagnetism is determined by the sum of the exchange parameters, *J* = *J*_*c*_ + *J*_*ab*_. We find that *J* increases with both electron and hole doping (Fig. 4d), which is largely due to the increasing ferromagnetic inter-plane exchange *J*_*c*_ (see Supplementary Figure 6g). The asymmetry between electron and hole doping is caused by the intra-plane exchange *J*_*ab*_, which increases with hole doping but decreases with electron doping (see Supplementary Figure 6g). The results for the exchange coupling *J* (Fig. 4d) indicate that the enhancement of ferromagnetism is more sensitive to the hole doping than to the electron doping, which is consistent with the experimental results for the ferromagnetic ordering temperature (Fig. 4b).

The effect of doping on the exchange parameters can be traced to the change in orbital overlap due to the Jahn–Teller distortions^39^. The Jahn–Teller effect leads Mn^3+^ ions bonded to oxygen to have one longer bond, *b*_l_, and one shorter bond, *b*_s_. The ratio of *b*_l_/*b*_s_ represents the extent of the distortion. As shown in Fig. 4d, upon electron or hole doping, the distortion is reduced compared the undoped system since Mn^2+^ and Mn^4+^ are not Jahn–Teller ions, causing Mn–O bonds to become more symmetric. This enhances ferromagnetism of LMO due to the increased contribution from ferromagnetic exchange between quarter-filled *e*_g_ orbitals and the decreased contribution from antiferromagnetic super-exchange between half-filled *t*_2g_ orbitals^39^. The enhancement of ferromagnetism with electron or hole doping is consistent with experimental^40,41^ and theoretical^42,43^ results for the chemically doped LMO.

### Discussion

Furthermore, it should be noted that the creation of the electron-doped ferromagnetic manganite was not obvious, being a challenging fundamental question full of controversy. Generally, the chemically doped *n*-type manganite always showed impurity phase and *p*-type behavior, and the conventional double or superexchange in electron-gated systems does not lead to ferromagnetism. Examples are La_1−*x*_Ce_*x*_MnO_3_^23,44,45^, La_1−*x*_Sb_*x*_MnO_3_^46^, and La_1−*x*_Te_*x*_MnO_3_^47^ etc, which always show impurity phase and *p*-type behavior. In addition, the conventional double-exchange/super-exchange in electron-gated systems does not lead to ferromagnetism, we therefore believe that the ambipolar ferromagnetic in manganite is unexpected in a hard doping situation. In addition, since there is no report on the AHE in electron-doped manganites yet, our observation completes the electron-doped region of the phase diagram of manganite thin film.

In conclusion, we have induced ambipolar ferromagnetism with a Curie temperature greater than 100 K in ultrathin films of LMO by bipolar gating. We found that the electrostatic doping of this material with either electrons or holes strengthens the ferromagnetic order in this compound, with a pronounced electron–hole asymmetry seen in the insulator-to-metal transition as well as the CMR effect. The ambipolar ferromagnet is a missing link between spintronics and semiconductor physics. Electric control of the charge of spin-polarized carriers offers an opportunity for future bipolar magnetic technology in strongly correlated electron systems.

## Methods

### Device fabrication

Three uc-thick LMO films were grown on TiO_2_-terminated STO (001) at 750 °C using pulsed laser deposition in an oxygen pressure of 10−^2^ mbar. The LMO growth is layer-by-layer and was monitored by in situ reflection high energy electron diffraction. After deposition, all samples were cooled down to room temperature in oxygen at the deposition pressure. The laser pulse (248 nm) energy density was 1.8 J cm^−2^ and the repetition rate was 1 Hz. Subsequently, the film was patterned into a Hall bar using photolithography.

### Electrical measurements

Carrier doping was realized by applying gate voltages (*V*_G_) on the double-layer capacitor formed by the ionic liquid. The ionic liquid used in the experiment was a small droplet of [*N*-diethyl-*N*-methyl-*N*-(2-methoxyethyl) ammonium bis (trifluoromethyl sulphonyl) imide] covering both the conducting channel and the gate electrode. The leakage current was less than 1 nA at a gate bias of *V*_G_ = ± 3 V. The electrical resistivity of the gated LMO films was measured using a Quantum Design Physical Property Measurement System (PPMS), a Keithley 2400 source measurement unit, and a Keithley 2182 nanovoltmeter from 300 K to 2 K in a magnetic field of 9 Tesla. Gate voltages were applied at 300 K between ionic liquid and LMO thin film to inject electrons or holes in LMO films, and the desired gate voltage was maintained throughout the whole transport measurement. Figure 4a is a re-plot of the data shown in Fig. 3a, b in MatLab using three standard plotting functions, namely Delaunay, trisurf, and shading. No manual averaging was done on the date and the horizontal stripes in Fig. 4a are generated due to the noise in resistance values in the raw data.

### STEM measurements

High-resolution cross-section STEM–HAADF imaging and EELS were performed using the JEOL-ARM200F microscope equipped with ASCOR aberration corrector and cold-field emission gun and operated at 200 kV. The cross-section TEM sample was prepared by focused ion beam with 30 kV Ga ions, followed by a 2 kV low voltage cleaning step. The HAADF images were acquired with a probe-forming aperture of 30 mrad and collection angle of 68–280 mrad. All HAADF images were filtered by radial Wiener filters. EELS spectra were recorded using a Gatan Quantum ER spectrometer with a 0.25 eV/channel energy dispersion.

### DFT calculations

Theoretical modeling of the orthorhombic *Pbnm* LMO was performed using density functional theory, the projected augmented wave method, and PBEsol pseudopotentials, as implemented in the Vienna ab initio simulation package. Internal coordinates were fully relaxed, assuming an experimental lattice constant of LMO and using the force convergence limit of 1 meV/atom. Correlation effects beyond generalized gradient approximation (GGA) were treated at a semi-empirical GGA + *U* level within a rotationally invariant formalism with *U* = 5 eV for the Mn *3d*-orbitals.

### Data availability

The authors declare that all other relevant data supporting the findings of the study are available in this article and in its Supplementary Information file. Access to our raw data can be obtained from the corresponding author upon reasonable request.

## Electronic supplementary material

Supplementary Information
