# Supplementary material for: Ambipolar ferromagnetism by electrostatic doping of a manganite
Source: Nat Commun. 2018 May 15;9:1897. doi: 10.1038/s41467-018-04233-5 (PMC5953920; doi:10.1038/s41467-018-04233-5)
Supplement: Supplementary file 1 — Supplementary Information [file 41467_2018_4233_MOESM1_ESM.pdf]

Supplementary Information

Ambipolar Ferromagnetism by Electrostatic Doping of a Manganite

Zheng et al.

Supplementary Note 1. Ionic liquid gating

In our study, the gate voltage, V_G, was applied at room temperature, and the voltage remains during the low-temperature measurement. Sheet resistance (R_S) measurement is performed during the cooling process from 300 to 2 K and the desired gate voltage was maintained throughout the whole process. The same gating procedures are maintained in all measurements.

Before characterizing our samples, a leakage current test was performed for every sample, with purposes of selecting functional devices and reducing leakage current. The leakage current test was always conducted using two sequential processes, namely a cooling process with V_G = - 1 V applied from 300 to 180 K and a warming process without V_G from 180 to 300 K. The leakage current remains in the nA range for good samples. It shall be noted that the leakage current is typically reduced after the two processes, possibly benefited from crystallization of water moisture in the ionic liquid.

We have completed a comprehensive characterization of the device stabilization and relaxation in a vacuum environment of 10^{-4} Torr at 300 K. Supplementary Figure 1 shows the temporal changes in the R_S when a constant gate voltage is applied at 300 K. With the V_G applied, the R_S reaches a nearly constant value after 30 min. Subsequently, the gate voltage is settled to zero, the R_S gradually returns to the initial value of R_S after a few hundred minutes at V_G = 0. Finally, R_S reaches a stable value that remains approximately constant, demonstrating that the electrostatic gating is the dominant effect.

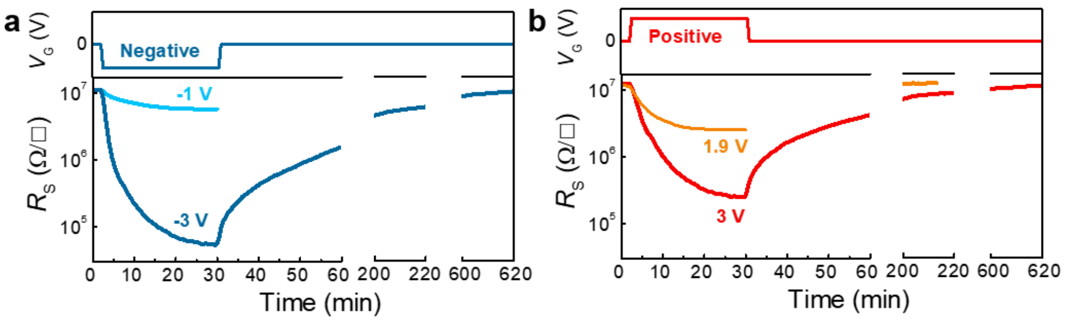

Supplementary Figure 1. Temporal changes in the sheet resistance (R_S) of an ionic liquid/3 unit cell (uc) $LaMnO_3/SrTiO_3$ (LMO/STO) device. The R_S as a function of time on applying a positive/negative voltage for 30 min and then setting the gate voltage to zero. All measurement were carried out inside a high vacuum chamber at a pressure of 10^{-4} Torr.

Supplementary Note 2. Electrical and morphology characterization before and after the gating

We conducted electrical and atomic force microscope measurements on 3 unit cell (uc) $LaMnO_3$ (LMO) samples before and after the gating measurements. Supplementary Figure 2a shows the R_S as a function of the temperature of an as-grown LMO before adding ionic liquid, exhibiting a semiconducting behaviour. Supplementary Figure 2b shows a typical atomic force microscope image of the surface of the as-grown 3 uc LMO. The surfaces of the samples are atomically flat, with a root-mean-square (RMS) surface roughness of ~ 0.16 nm. Subsequently, the temperature-dependent R_S of the LMO thin film was measured with positive and negative 3 V V_G across ionic liquid at 300 K. Supplementary Figure 2c shows that the -3 V induces a clear metal-to-insulator transition, and the R_S recovers original state after removing the gate voltage. Then, a 3 V V_G was applied and the device shows consistent transport phenomena. In the case of structural defects, such as cationic defects or oxygen vacancy, the R_S will not immediately revert to the original state, due to the extremely low mobility of the defects around or below room temperature and the dramatic impact on R_S from the defects.

After the ionic liquid gating experiments, we removed the ionic liquid by sonicating the sample in acetone for about 10 min. Then another atomic force microscope image was taken after washing the sample in alcohol. Supplementary Figure 2d shows that no measurable changes in topography were observed after gating. The RMS surface roughness remains ~ 0.16 nm. Since etching due to ionic liquid would produce a rough surface, this indicates that etching of the material is negligible.

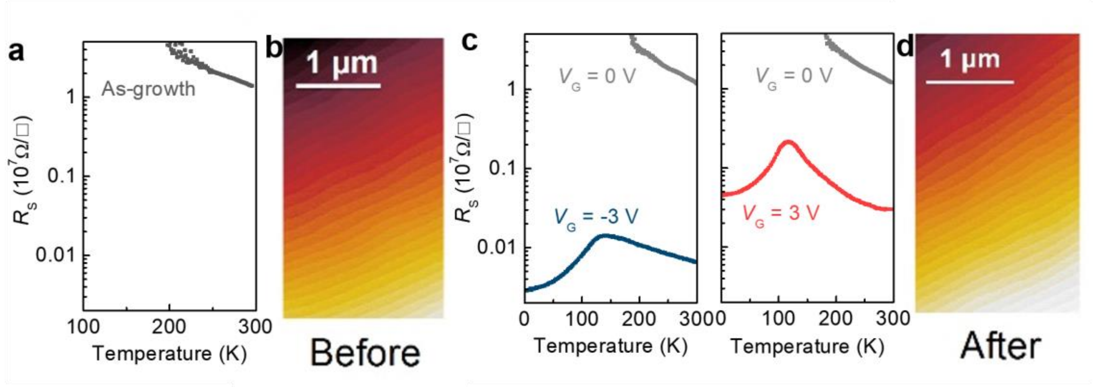

Supplementary Figure 2. Electrical and surface characterization before and after the gating experiment. The R_S of LMO as a function of the temperature (a) and the atomic force microscope image of LMO (b) before applying any gate voltage. (c) The R_S as a function of the temperature of LMO under positive and negative 3 V gate voltage. (d) The atomic force microscope image of LMO without ionic liquid after two electrical measurements with positive and negative 3 V V_G applied.

Supplementary Note 3. Experimental reproducibility

The ionic liquid gating experiment and corresponding Hall measurement were repeated on other samples. Supplementary Figure 3a,b demonstrates the function between R_S and temperature on another LMO sample. The behavior of the sample is in a good agreement with the results presented in the main text, demonstrating reproducibility of the ionic liquid gating experiment.

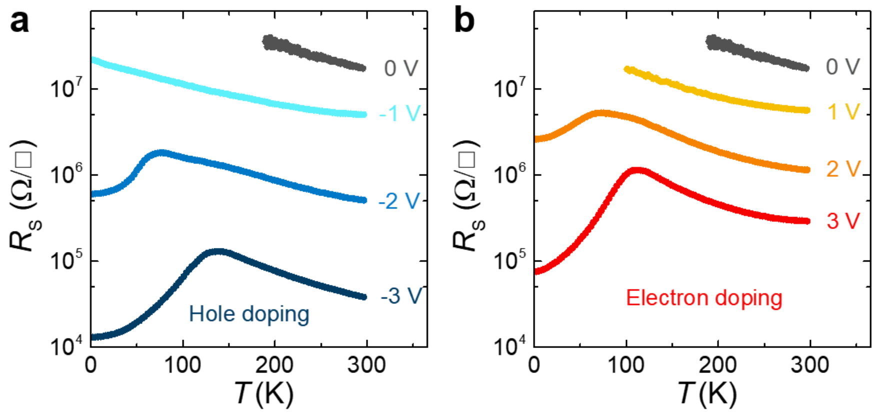

Supplementary Figure 3. Reproducibility of ionic liquid gating experiment. Temperature dependent R_S for (a) electron- and (b) hole-doped LMO, respectively.

Supplementary Note 4. Cooling and warming runs for the resistance measurements

In manganites competing phases, namely ferromagnetic conducting phase (FM-C) and charge-ordered insulating phase (CO-I), may coexist at the mesoscopic length scale. Typically, the presence of thermal hysteresis serves as evidence of phase separation in manganites and its absence may suggest a single-phase magnetic state in our samples. For example, significant hysteretic behavior were observed in $Pr_{0.65}(Ca_{0.75}Sr_{0.25})_{0.35}MnO_3$ (PCSMO)[1], by J. Laurembam *et al.*, $Pr_{1-x}Sr_xMnO_3$ (PSMO)[2] by T. Hatano et al., and $La_{1-x-y}Pr_yCa_xMnO_3$ (LPCMO)[3] by M. Uehara *et al.*

As already shown in the Supplementary Figure 1, we find no signature of thermal hysteresis in our resistivity data when cooling and heating the sample with both positive and negative 3 V V_G applied across the ionic liquid.

In addition, studying phase separation from first-principles would be very valuable. However, the task of taking into account non-uniform doping and local structural distortions in first-principles calculations, which also need to include electron-electron correlation effects, to explore competing phases in strongly correlated oxide materials is enormously complicated. Thus far, there is no clear understanding how even to approach this problem yet. The only attempts are analysis based on model Hamiltonians[4,5], which were used to map out the spatial distribution and size of the competing phases on the mesoscopic scale. Therefore, more work is needed to understand the possibility of competing phases in our samples, which we cannot completely exclude.

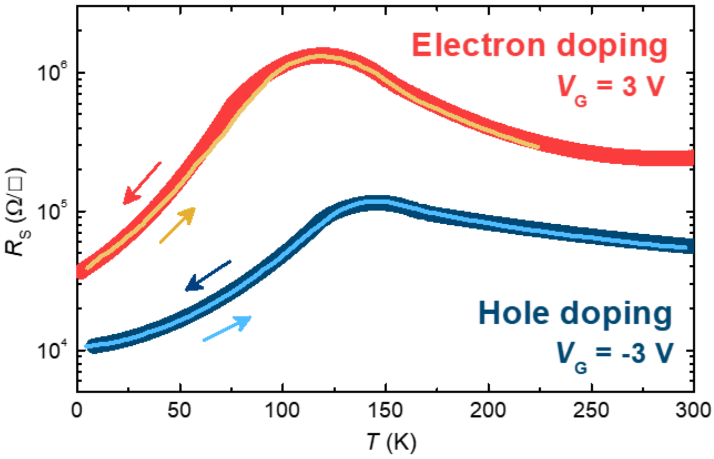

Supplementary Figure 4. Cooling and warming measurement of resistance for both electron- and hole-doped $LaMnO_3$. The electron- and hole-doping were realized by applying +3 and -3 V V_G across the ionic liquid.

Supplementary Note 5. Hall effect measurement

The Hall effect measurement was performed at different temperatures in a magnetic field up to 9 Tesla. The Hall conductivity (σ_{xy}) is defined by the expression of ρ_{xy}/ρ_{xx}^2, where ρ_{xx} and ρ_{xx} are transverse and longitudinal resistivity, respectively. The σ_{xy} of CMR material can usually be expressed as

$$\sigma_{xy} = \sigma_{xy}^{OHE} + \sigma_{xy}^{AHE} \quad (1)$$

where σ_{xy}^{OHE} and σ_{xy}^{AHE} are the ordinary and anomalous Hall conductivity, respectively. At room temperature, 300 K, the slope of ρ_{xy} is dominated by the anomalous Hall effect (AHE) contribution. Hence, the sign of the ρ_{xy} at 300 K represents reversed type of carriers for both electron- and hole-doping gating cases[6,7].

It is therefore inappropriate to conclude the sign and density of carrier at room temperature. At low temperature, the ρ_{xy} has a measureable and stronger ordinary Hall effect (OHE) contribution at high field. The OHE can be written as $R_H = 1/n(p)e$, where R_H is the ordinary Hall coefficient, e is elementary charge, and $n(p)$ is carrier density for electron(hole). In Fig. 2d of the main text, the Hall carrier density of LMO was obtained by applying the definition of OHE and was based on the gate voltage dependent ρ_{xy} shown in Supplementary Figure 5.

This is The AHE contribution is thought to originate from the skew scattering ($\sigma_{xy}^{AHE-skew}$), side jump processes (σ_{xy}^{AHE-sj}), and intrinsic contribution ($\sigma_{xy}^{AHE-int}$), which dominant at different regimes of longitudinal conductivity (σ_{xx}). As a function of σ_{xx} for diverse materials, the three broad regimes are (i) a high conductivity regime [10^6 $(\Omega\ \text{cm})^{-1} < \sigma_{xx}$] in which AHE are due to dominant skew scattering and normal Hall effect can be visible; (ii) in intermediate regime [10^4 $(\Omega\ \text{cm})^{-1} < \sigma_{xx} < 10^6$ $(\Omega\ \text{cm})^{-1}$] where AHE is independent of σ_{xx} due to intrinsic contribution; and (iii) a bad-metal regime [$\sigma_{xx} < 10^4$ $(\Omega\ \text{cm})^{-1}$] where AHE changes dramatically with changing σ_{xx}. In particular, the intrinsic mechanisms occur in magnetic materials with strong spin-orbit coupling, such as oxides and diluted magnetic semiconductors (DMSs). Manganites, including the gated 3 uc LMO, have a σ_{xx} between 10^{-1} to 10^4 $(\Omega\ \text{cm})^{-1}$ and are in the bad-metal regime.

Unfortunately, direct measurement of magnetism in LMO is prohibited by two major technical challenges, namely (a) the extremely weak signal from the limited volume of our 5 uc-thick, 300 μm-long and 50 μm-wide LMO, and (b) spurious signal from applied current, gold electrode, and contamination in ionic liquid.

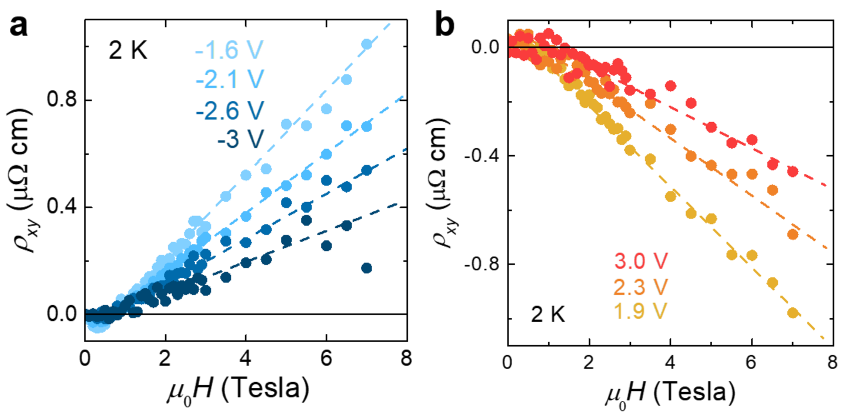

Supplementary Figure 5. ρ_{xy} of (a) hole- and (b) electron-doped LMO under various gate voltages at 2 K.

Supplementary Note 6. First-principles calculations

Theoretical modelling of the orthorhombic *Pbnm* LMO was performed using density functional theory, the projected augmented wave method, and PBEsol pseudopotentials[8], as implemented in the Vienna *ab initio* simulation package[9]. Correlation effects beyond generalized gradient approximation (GGA) were treated at a semi-empirical GGA+U level within a rotationally invariant formalism with U = 5 eV on Mn *3d*-orbitals[10]. We used 340 eV kinetic energy cutoff and 7×7×5 k-points for Brillouin zone integration. Calculations were performed using a $\sqrt{2}\times\sqrt{2}\times 2$ pseudocubic unit cell, which contains 20 atoms (Supplementary Figure 6a). To simulate epitaxial growth of LMO on $SrTiO_3$ (001) (STO) substrate, the in-plain pseudocubic lattice constant was constrained to the theoretical lattice constant of STO, which was found to be $a = b = 3.90$ Å. The out-of-plane lattice constant c and all internal atomic coordinates were fully relaxed for each doping level and magnetic configuration, using the force convergence limit of 1 meV/atom. The constraint of the in-plain lattice constant led to LMO being biaxially compressed by about 2%, resulting in $b/a = 1$ and c/a =1.41, as compared to the bulk values of b/a = 1.03 and c/a = 1.39. The bipolar doping of LMO was performed by adding electrons/holes to the system and neutralizing the total charge by jellium background.

To explore magnetism of LMO as a function of doping, we computed the total energies of different magnetic configurations: ferromagnetic (FM), A-type antiferromagnetic (A-AFM), C-type antiferromagnetic (C-AFM), G-type antiferromagnetic (G-AFM), and E-type antiferromagnetic (E-AFM), as shown in Supplementary Figure 6a-e, respectively (note that the unit cell was doubled for the E-AFM configuration). We found that, at zero doping, the ground state of the biaxially strained LMO is FM in contrast to unstrained bulk LMO, which is known to have an A-AFM ground state. This is explained by the reduced Jahn-Teller distortion of the biaxially strained LMO, as discussed below. Magnetic exchange parameters were obtained by mapping the total energies of different magnetic configurations to the anisotropic Heisenberg Hamiltonian, $H = -\sum_{ij} j_{ij} S_i \cdot S_j$, where j_{ij} is the exchange interaction parameters between neighboring Mn ions with localized moments S_i and S_j. Taking into account of the first and the second nearest neighbors, the resulting energies are given by[11]

$$E_{FM} = -4j_{ab}^1 - 2j_{ab}^2 - 2j_c^1 - 8j_c^2 \quad \mathbf{(2)}$$

$$E_{A-AFM} = -4j_{ab}^1 - 2j_{ab}^2 + 2j_c^1 + 8j_c^2 \quad \mathbf{(3)}$$

$$E_{C-AFM} = 4j_{ab}^1 - 2j_{ab}^2 - 2j_c^1 + 8j_c^2 \quad \mathbf{(4)}$$

$$E_{G-AFM} = 4j_{ab}^1 - 2j_{ab}^2 + 2j_c^1 - 8j_c^2 \quad \textbf{(5)}$$

$$E_{E-AFM} = 2j_{ab}^2 + 2j_c^1 \quad \textbf{(6)}$$

where j_{ab}^1 and j_{ab}^2 are the first and the second nearest-neighbor intra-plane exchange interactions and j_c^1 and j_c^2 are the first and the second nearest-neighbor inter-plane exchange interactions. Taking into account of a reference energy, this system of five linear equations contains five unknowns and can be uniquely solved with respect to the exchange constants. In a layered system, it is convenient to express the exchange in terms of total intra-plane exchange J_{ab} and total inter-plane exchange J_c. Considering the number of the first and the second nearest neighbors, the total intra-plane exchange coupling is given by $J_{ab} = 4j_{ab}^1 + 2j_{ab}^2$. Similarly, the total inter-plane exchange is $J_c = 2j_c^1 + 8j_c^2$.

Supplementary Figure 6h shows the calculated nearest-neighbor exchange parameters j_{ab}^1, j_{ab}^2, j_c^1 and j_c^2, as a function of doping. As seen in the figure, j_{ab}^1, j_c^1 and j_{ab}^1 are positive (ferromagnetic), while j_{ab}^2 is negative (antiferromagnetic). With doping, j_{ab}^1 and j_c^1 increases, j_c^2 remains nearly constant, while j_{ab}^2 decreases. The overall intra-plane exchange J_{ab} in the first nearest neighbors involves twice more than that in the second nearest neighbors, which leads to a FM ordering in the plane (Supplementary Figure 6g). The intra-plane exchange J_{ab} increases with hole doping but decreases with electron doping, due to the stronger reduction of j_{ab}^2 with electron doping as compared to hole doping (see Supplementary Figure 6h). The total inter-plane exchange J_c is largely determined by the exchange coupling between the first nearest neighbors j_c^1, and is strongly enhanced with both electron and hole doping (see Supplementary Figure 6g). Hence, the enhanced inter-plane exchange strengthens the ferromagnetic state of LMO as observed experimentally.

Jahn-Teller distortions lead to Mn ions bonded to oxygen having one longer bond, b_l, and one shorter bond, b_s. The ratio of b_l/b_s represents the extent of the Jahn-Teller distortion. We found that in biaxially strained A-AFM LMO, b_l/b_s is reduced to ≈1.09 from b_l/b_s≈1.13 of unconstrained relaxed bulk. With doping, the short bond increases and long bond decreases, thereby decreasing b_l/b_s further, as shown in Fig. 4d of the main text. According to Soloyev *et al.*[12], when b_l/b_s < 1.12, the total inter-plane exchange becomes FM, which is further enhanced upon reduced distortions. This is consistent with our result at zero doping (b_l/b_s≈1.09) and at finite doping (Fig. 4d of the main text), where b_l/b_s is reduced down to about 1.07 or 1.073 with the doping of 0.1 or –0.1 e/Mn.

The orbital ordering of biaxially strained LMO was found to be similar to that known

for bulk LMO. Supplementary Figure 6f shows the charge density contour of LMO in the *a-b* plane, which reveals a checker-board-type orbital ordering in that plane, similar to that found previously for bulk LMO[12]. This ordering is maintained when the system is homogeneously doped.

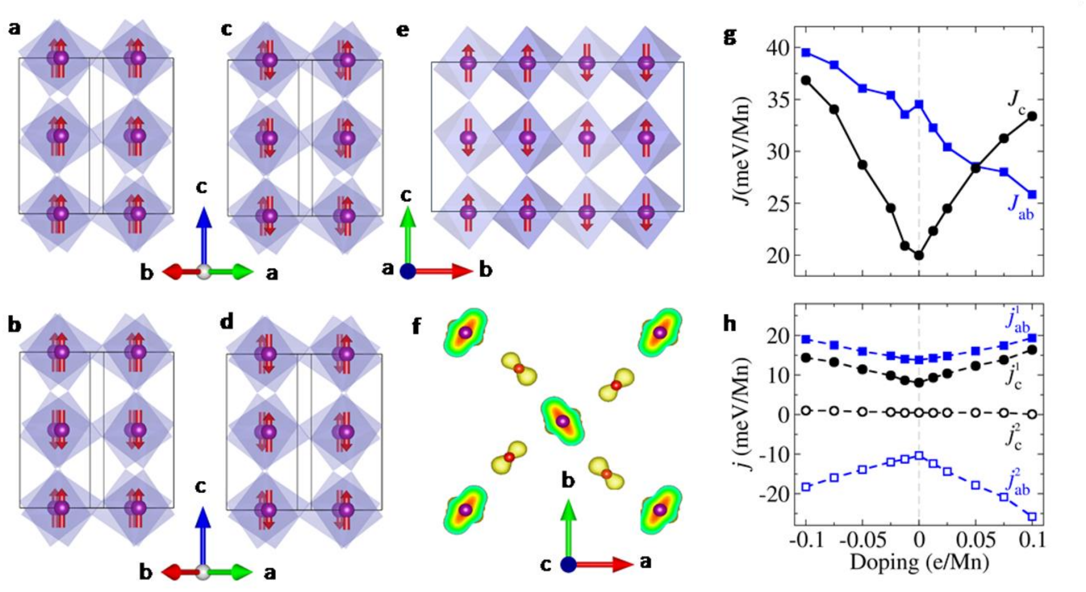

Supplementary Figure 6. Magnetic structures, orbital ordering, and doping dependent exchange parameters in biaxially-strained LMO. (a) Ferromagnetic, (b) A-type antiferromagnetic, (c) C-type anti-ferromagnetic, (d) G-type antiferromagnetic, and (e) E-type antiferromagnetic ordering configurations on a unit cell of orthorhombic LMO. (f) Charge density contour of LMO in the a-b plane, showing a checker-board-type orbital ordering. (g) Calculated intra-plane (J_{ab}) and inter-plane (J_c) exchange coupling parameters as a function of doping. (h) Calculated the first (j_{ab}^1, j_c^1) and the second (j_{ab}^2, j_c^2) nearest neighbor intra-plane (indicated by index a,b) and intra-plane (indicated by index c) exchange coupling parameters as a function of doping. Positive and negative signs designate ferromagnetic and antiferromagnetic exchange, respectively.

Supplementary Note 7. Structural characterization

First, we imaged 3 uc LMO deposited on STO substrate. Supplementary Figure 7a shows a high-angle annular dark field (HAADF) image of the sample along the [010] zone axis. Supplementary Figure 7b shows the EELS of the uncapped 3 uc LMO. In the ADF image, the contrast of the outermost unit cell of LMO becomes gradually blurry. But, the EELS signal from the outermost unit cells of LMO is still visible. This

indicates that the outermost unit cell of LMO becomes partially amorphous possibly due to the damage by the focused ion beam during the STEM sample preparation.

Therefore, in order to correctly characterize the 3 uc LMO and avoid the damage during the STEM sample preparation, we added a STO capping layer to protect the surface of LMO and performed STEM-EELS using the same conditions (see Fig. 1 of Main Text).

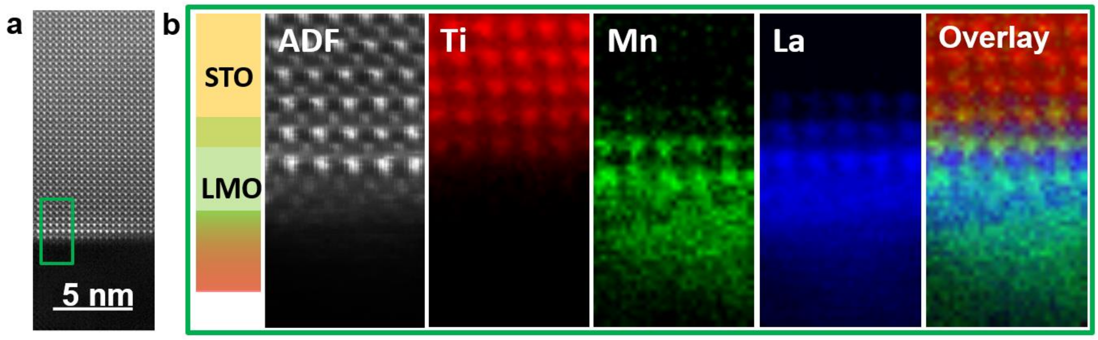

Supplementary Figure 7. Structural characterization on uncapped 3 uc LMO deposited on the STO substrate. (a) HAADF image of uncapped 3 uc LMO. The outermost unit cell is damaged and cannot give a clear picture. The scale bar is 5 nm. (b) EELS of uncapped 3 uc LMO deposited on the STO substrate. The intermixing between Mn and Ti is about 1 uc. The corresponding EELS region is indicated in the HAADF image by the green box in the panel (a).

Supplementary Note 8. Full field effect transistor (FET) characterization measurements

Supplementary Figure 8 presents basic characteristics of an ionic liquid/$LaMnO_3$ device at 300 K under a small DC source-drain bias (V_{SD} = 100 mV). Supplementary Figure 8a shows a typical transfer curve of the device with a negligible (~1 nA) leakage current (I_G) (Supplementary Figure 8b). The output curves of the ionic liquid/$LaMnO_3$ device under both positive and negative gate voltages are shown in Supplementary Figure 8c,d.

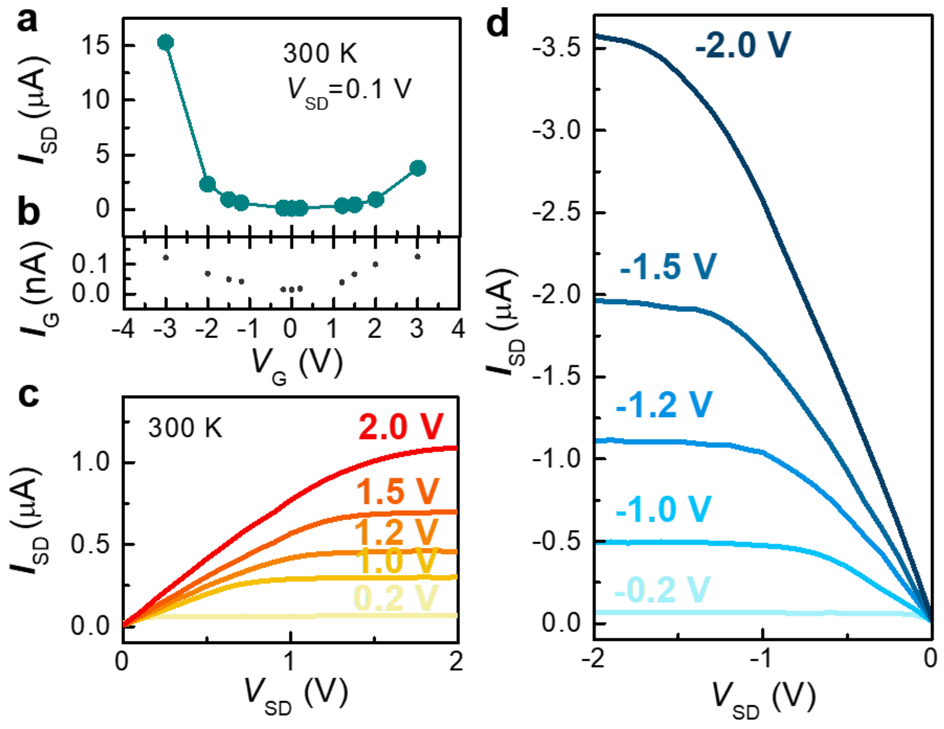

Supplementary Figure 8. Basic characteristics of an ionic liquid/$LaMnO_3$ device at 300 K. (a) Transfer characteristic: I_{SD}-V_G at V_{SD} = 100 mV. (b) The corresponding leak current, I_G, measured for the transfer curve, which is shown in the panel (a). (c,d) I_{SD}-V_{SD} output curve with applied V_G from 0 to -2 and 0 to 2 V.

Supplementary Reference

1. Lourembam, J., Wu, J., Ding, J., Lin, W. and Wu, T. Electric field tuning of phase separation in manganite thin films. *Phys. Rev. B* **89,** 014425 (2014).
2. Hatano, T. *et al.* Gate control of electronic phases in a quarter-filled manganite. *Sci. Rep.* **3,** 2904 (2013).
3. Uehara, M., Mori, S., Chen, C. H. & Cheong, S.-W. Percolative phase separation underlies colossal magnetoresistance in mixed-valent manganites. *Nature* **399,** 560–563 (1999).
4. Burgy, J., Mayr, M., Martin-Mayor, V., Moreo, A. & Dagotto, E. Colossal effects in transition metal oxides caused by intrinsic inhomogeneities. *Phys. Rev. Lett.* **87,** 277202 (2001).
5. Burgy, J., Moreo, A. & Dagotto, E. Relevance of cooperative lattice effects and stress fields in phase-separation theories for CMR manganites. *Phys. Rev. Lett.* **92,** 097202 (2004).
6. Malfait, M. *et al.* Sign inversion of the high-field Hall slope in epitaxial

$La_{0.5}Ca_{0.5}MnO_3$ thin films. *Phys. Rev. B* **68,** 132410 (2003).
7. Nair, S. *et al.* Hall effect in heavy fermion metals. *Adv. Phys.* **61,** 583–664 (2012).
8. Perdew, J. *et al.* Restoring the density-gradient expansion for exchange in solids and surfaces. *Phys. Rev. Lett.* **100,** 136406 (2008).
9. Kresse, G. & Furthmüller, J. Efficiency of ab-initio total energy calculations for metals and semiconductors using a plane-wave basis set. *Comput. Mater. Sci.* **6,** 15–50 (1996).
10. Rodriguez-Carvajal, J. *et al.* Neutron-diffraction study of the Jahn-Teller transition in stoichiometric $LaMnO_3$. *Phys. Rev. B* **57,** R3189–R3192 (1998).
11. Yamauchi, K. *et al.*, Magnetically induced ferroelectricity in orthorhombic manganites: Microscopic origin and chemical trends. *Phys. Rev. B* **78**, 014403 (2008).
12. Solovyev, I. *et al.*, Crucial role of the lattice distortion in the magnetism of $LaMnO_3$. *Phys. Rev. Lett* **76**, 4825 (1996).
